# C-Peptide Levels Predict the Effectiveness of Dipeptidyl Peptidase-4 Inhibitor Therapy

**DOI:** 10.1155/2016/4509603

**Published:** 2016-11-02

**Authors:** Sevin Demir, Sule Temizkan, Mehmet Sargin

**Affiliations:** ^1^Department of Family Medicine, Eleskirt Public Hospital, 04600 Agri, Turkey; ^2^Department of Endocrinology and Metabolic Diseases, Kartal Dr. Lutfi Kirdar Training and Research Hospital, 34890 Istanbul, Turkey; ^3^Department of Family Medicine, Medeniyet University Goztepe Training and Research Hospital, 34722 Istanbul, Turkey

## Abstract

*Background*. Our aim was to define the conditions that affect therapeutic success when dipeptidyl peptidase-4 (DPP-4) inhibitor is added to metformin monotherapy.* Materials and Methods*. We reviewed the medical records of 56 patients who had received DPP-4 inhibitor as an add-on to metformin monotherapy and evaluated their response in the first year of therapy. Fasting blood glucose (FBG), HbA1c, C-peptide, and weight of the patients were recorded at 3-month intervals during the first year of treatment.* Results*. Patients who added DPP-4 inhibitor to metformin monotherapy had significant weight loss (*P* = 0.004) and FBG and HbA1c levels were significantly lowered during the first 6 months (both *P* < 0.001). Baseline levels of C-peptide were predictive for success of the treatment (*P* = 0.02), even after correction for confounding factors, for example, age, gender, or BMI (*P* = 0.03). Duration of diabetes was not a predictor of response to treatment (*P* = 0.60).* Conclusion*. Our study demonstrates that in patients having inadequate glycemic control, the addition of a DPP-4 inhibitor as a second oral agent to metformin monotherapy provides better glycemic control, protects *β*-cell reserves, and does not cause weight gain. These effects depend on baseline C-peptide levels.

## 1. Introduction

Type 2 diabetes mellitus (T2DM) is a chronic disease arising from both environmental and genetic factors. T2DM is characterized by disrupted secretory activity in pancreatic *β*-cells, resistance to insulin action in peripheral tissues, or both together. This progressive loss of glycemic control results in chronic hyperglycemia. New treatment options for T2DM are needed because current treatment regimens have dose-dependent limitations and side effects, such as weight gain [1–3].

It has been shown that glucose administered orally produces a higher insulin response compared to the same amount of intravenous glucose, and the difference is attributable to the effects of incretins. Two hormones are believed to be responsible for incretin effects: glucose-dependent insulinotropic peptide (GIP) and glucagon-like peptide-I (GLP-I) [1]. GLP-1 regulates blood glucose mainly by acting on pancreatic islet cells via mechanisms that include the stimulation of insulin secretion [1]. GLP-1 preserves beta cell by inhibiting apoptosis, although increase in beta cell mass has not been observed with any GLP-1 based therapy in man, even in rodents [4]. GLP-1 also inhibits glucagon secretion and suppresses gastric emptying, gastric secretion, and exocrine pancreatic secretion. It has also been reported to have cardioprotective and neuroprotective effects [1]. GLP-1 and GIP are secreted in response to enteral nutrient loads but are rapidly cleaved by dipeptidyl peptidase-4 (DPP-4). This finding of rapid enzyme cleavage prompted the development of two successful therapeutic strategies for T2DM: the use of DPP-4 resistant GLP-1 receptor agonists and DPP-4 inhibitors [3, 5].

There seems to be DPP-4 inhibitors lower postprandial blood glucose by preventing the rapid degradation of incretins in early-stage T2DM patients who have enough pancreatic insulin reserve [6–8]. There is frequently a perception that treatment with these agents may be less efficacious with increasing disease progression, due to loss of beta cell function and increasing insulin resistance. Although, a study has shown that vildagliptin added on metformin therapy was efficacious independent of insulin resistance stage, body mass index (BMI), and disease duration and duration of prior metformin use [9].

C-peptide is a marker that can be used to indicate the level of endogenous insulin reserve and beta cell function [8]. In this study, our aim was to investigate the responses obtained with the addition of DPP-4 inhibitor therapy to metformin monotherapy and determine whether or not there was an association between baseline levels of C-peptide levels and the effectiveness of 1 year of DPP-4 inhibitor treatment. We also investigated whether DPP-4 inhibitor therapy would result in any change in C-peptide levels.

## 2. Materials and Methods

### 2.1. Subjects

We studied 56 patients with T2DM, whose HbA1c was ≥7% and who had used metformin monotherapy at least for 1 year and sitagliptin therapy added-on. Patients having any change in their treatment during a 1-year follow-up or those who were not compatible with treatment for any other reason were excluded from the study. We also excluded patients who had used insulin and/or sulfonylurea or other oral antidiabetic drugs; those who had a history of malignancy, pancreatitis, chronic kidney disease, chronic liver disease, or gastrointestinal surgery; and those who were using glucocorticoid therapy. The study was conducted in Dr. Lutfi Kirdar Kartal Research and Training Hospital, Diabetes Unit, between January 2009 and January 2015. Patient medical records and laboratory data were reviewed during the first year of DPP-4 inhibitor therapy.

The study was conducted in agreement with the Declaration of Helsinki II. Kartal Dr. Lutfi Kirdar Training and Research Hospital Ethical Committee approved the study protocol. Informed consent was not required because of the retrospective nature of our study.

### 2.2. Study Design

Demographic characteristics including height, weight, duration of diabetes, comorbidities, and concomitant medications were obtained from patient files. Height and weight were measured four times in 3-month intervals during a 1-year period. The body mass index (BMI) was calculated as weight in kilograms divided by height in square meters.

We assessed the response to DPP-4 inhibitor therapy using change from baseline in HbA1c after 6 or 12 months of glucose-lowering therapy (ΔHbA1c). Good responders were defined as ΔHbA1c decreasing by more than 1% and/or HbA1c values falling below 7.0%.

FBG, HbA1c, and C-peptide levels were measured at 3-month intervals. Plasma venous glucose was measured using the hexokinase method. HbA1c was assayed by high-performance liquid chromatography. Serum insulin levels were measured by the immunoassay method (Abbott Diagnostics, USA).

### 2.3. Statistical Analysis

Data are expressed as mean ± standard deviation (SD). Patient glucose parameters and weights at baseline, 6 months, and 12 months were compared using the paired *t*-test. Predictors of success in the sixth month of treatment were analyzed by logistic regression. The results were adjusted for age, gender, and BMI (Model 2).

## 3. Results

In total, 56 subjects with T2DM were included in the study. A total of 35 patients were females and 21 were males. The average age was 61 ± 9 years, and the average duration of diabetes was 13 ± 5 years. The average baseline HbA1c was 7.7%, and average C-peptide levels were 2.4 ± 0.8 ng/mL (Table 1).

Patients who added DPP-4 inhibitor to their metformin monotherapy showed statistically significant weight loss (*P* = 0.004) (average = 1 kg) after 6 months of treatment, but no additional weight loss during the second 6 months of treatment (*P* = 0.86). Similarly, the FBG and HbA1c levels were significantly decreased during the first 6 months of treatment (both *P* < 0.001), and the decrease was maintained during the second 6-month period with no further decrease (*P* = 0.25, *P* = 0.38, resp.). Thus, the effects of DPP-4 inhibitors occurred during the first 6 months of treatment and remained stable thereafter (Table 2).

The C-peptide levels at baseline and at the end of the first year are shown in Table 3. After 12 months of treatment, the C-peptide levels were significantly higher (*P* < 0.001).

Predictors of success during the first 6 months of treatment were evaluated in Table 4. Baseline levels of C-peptide were a statistically significantly predictor of success of the treatment (*P* = 0.02), even after correction for confounding factors such as age, sex, and BMI (*P* = 0.03). The duration of diabetes was not a predictor of response to treatment (*P* = 0.60).

## 4. Discussion

T2DM is a chronic disease characterized by disruption of insulin secretion by pancreatic beta cells, insulin resistance in peripheral tissues, or both together. T2DM is becoming a bigger health problem day by day. Current guidelines are in agreement with metformin as an initial treatment unless there is a contraindication, but recommendations vary regarding add-on agents for patients who do not achieve glucose-lowering goals with metformin monotherapy. Since the etiology of T2DM has a link with obesity, it is important that an additional agent should not cause weight gain. In this study, we report that DPP-4 inhibitors do not cause weight gain and they act to preserve beta cell function; we also report that pretreatment levels of C-peptide levels predict success of the add-on treatment in patients already using metformin monotherapy.

In previous reports where homeostasis model assessment of beta-cell (HOMA-*β*) and proinsulin to insulin ratio has been used to assess beta cell function, in our study, we have demonstrated beta cell function using C peptide levels, because, in our clinical practice, the measurements of C-peptide are more easily accessible.

D'Alessio et al. have reported the achievement of good glycemic control in 41 patients with T2DM and HbA1c values ranging from 6.5 to 7.5 who used either diet therapy or metformin monotherapy for 6 months prior to being randomized to vildagliptin or placebo for a period of 12 weeks. The authors reported that vildagliptin provided a significant reduction in HbA1c and increased acute insulin response as well as acute C-peptide response to glucose [10].

A study by Mari et al., examining 20 patients with T2DM, showed that vildagliptin improves the rate of insulin secretion and glucose sensitivity in pancreatic beta cell [11]. Similarly, in our study reports, a significant reduction in HbA1c and significant increase in C-peptide levels at the end of one year of treatment were obtained.

Brazg et al. studied 28 patients with T2DM who had been treated with metformin monotherapy randomized and were then randomized to sitagliptin or placebo for 28 days. A 24 h monitoring showed that the C-peptide levels in the sitagliptin group were low after lunch, high after dinner, and average over the course of the entire day and there was no difference between the two groups [12].

A study by Henry et al. randomized 36 patients who had used saxagliptin or placebo for a period of 12 weeks. The investigators evaluated insulin secretion by the intravenous-oral hyperglycemic clamp test. Consistent with other studies, the speed of the insulin secretion was significantly increased with the DPP4 inhibitors but showed no change in C-peptide levels [13].

Patients with impaired fasting glucose (IFG) are known to have low GLP-1 levels, but it is unknown whether this condition is caused directly by IFG itself. Bock et al. studied 22 patients with IFG who had been treated with sitagliptin or placebo for 8 weeks. The patients were fed the same standardized meal at the beginning and end of treatment. No significant difference was observed in FBG, postprandial plasma glucose, C-peptide, or insulin levels. Low incretin concentrations are unlikely to be involved in the pathogenesis of IFG [14].

We know that incretins are effective after oral intake; therefore, using DPP-4 inhibitors in patients who have only high fasting glucose levels could not make a significant difference. In addition, a study conducted by El-Ouaghlidi et al. showed no improvement in insulin response with DPP-4 inhibitors in healthy subjects [15]. In our study, we observed a decrease in the FBG levels in the first 6 months but this could not continue through the second 6 months.

In a study conducted by Derosa et al., 171 patients with T2DM were instructed to take metformin for 8 months. Then, they were randomly assigned to add vildagliptin or placebo for 12 months. After 12 months, both groups had lost significant weight, and the group using vildagliptin lost more weight than the other group (−5.8 versus −5.1). After 12 months of treatment, vildagliptin + metformin gave a better decrease of HbA1C (−1.8% versus −1.2%). In this study, several parameters were used to show beta cell function: HOMA-*β*, fasting plasma proinsulin, proinsulin/fasting plasma insulin, C-peptide, C-peptide response to glucose, and C-peptide response to arginine. As a result of this study, it was shown that the addition of vildagliptin to metformin gave a better improvement of beta cell function compared to metformin alone [16].

Oh et al. reviewed the medical records of 477 patients who had taken sitagliptin or vildagliptin longer than 40 weeks. The response to DPP-4 inhibitors was evaluated with HbA1c change after therapy (ΔHbA1c). In this study, good response to DPP-4 was defined as ΔHbA1c > 1.0% and poor response as ΔHbA1c < 0.5%. According to the sex-adjusted partial correlation analysis, duration of diabetes, FBG, HbA1c, C-peptide, and creatinine concentrations were associated with ΔHbA1c. They found that a better response to DPP-4 inhibitors would be expected in patients with T2DM who had higher baseline HbA1c and creatinine levels with shorter duration of diabetes [17].

In summary, our study showed that while beginning a DPP-4 inhibitor as a second oral agent to insufficient glycemic control with metformin monotherapy, it provides better glycemic control, patients do not gain weight, and it protects beta cell reserves. These effects depend on the baseline C-peptide levels.

## Figures and Tables

**Table 1:** General characteristics of the subjects (*n* = 56).

Age (year)	61 ± 9
Gender (F/M)	35/21
BMI (kg/m^2^)	31.0 ± 5.3
Duration of diabetes (year)	13 ± 5
FBG (mg/dL)	162 ± 25
HbA1c (%)	7.7 ± 0.5
C-peptide (ng/mL)	2.4 ± 0.8

**Table 2:** Comparison of the weights and glucose parameters in subjects at baseline, sixth month, and twelfth month.

	Baseline	6th month	12th month	*P* ^a^	*P* ^b^	*P* ^c^
Weight (kg)	82 ± 14	81 ± 14	81 ± 14	0.004	0.01	0.86
FBG (mg/dL)	162 ± 25	149 ± 33	154 ± 33	<0.001	0.09	0.25
HbA1c (%)	7.7 ± 0.5	7.0 ± 0.6	7.1 ± 0.8	<0.001	<0.001	0.38

Paired *t*-test

*P* ^a^: comparison of the baseline and sixth month

*P* ^b^: comparison of the baseline and twelfth month

*P* ^c^: comparison of the sixth month and twelfth month.

**Table 3:** Comparison of C-peptide levels in the baseline and twelfth month.

	Baseline	12th month	*P*
C-peptide (ng/mL)	2.4 ± 0.8	2.9 ± 1.0	<0.001

**Table 4:** Predictors of treatment success at sixth month.

	Model 1		Model 2	
	95% CI	*P*	95% CI	*P*
Baseline C-peptide	2.5 (1,15–5,77)	**0.02**	2.5 (1,04–6,09)	**0.03**
Duration of diabetes	1.0 (0,92–1,20)	0.41	1.0 (0,90–1,18)	0.60
Age			1.0 (0,94–1,09)	0.66
Gender			1.9 (0,49–7,59)	0.34
BMI			1.0 (0,89–1,18)	0.71

Logistic regression analysis.
